# Retention of a Dead Fœtus by a Cow

**Published:** 1892-03

**Authors:** D. P. Frame

**Affiliations:** Colorado Springs, Col.


### RETENTION OF A DEAD FŒTUS BY A COW.

By D. P. Frame.

The following case being of rare occurrence I deem it of sufficient interest to report for the benefit of the profession. On November 28, 1891, while attending some stock on the ranch of Messrs. Erich and White, near this city, I was requested to examine one of their best Holstein cows to determine whether or not she was in calf, she being then two months past her time and showing no signs of calving. An examination was made *per rectum*, but I was unable to decide positively whether there was a fœtus there or not, as I could feel no movement, nor could I make out any definite form of a fœtus, though there appeared to be a hard mass in the uterus. The cow was in perfect health and good condition, but had made no preparation for calving at the usual time, nor did she come in heat.

On January 12th, 1892, I was again requested to examine this cow as she showed no signs of calving, but was steadily improving in condition, and being valuable as a breeder, her owners were desirous of having her services if possible. On the above date I made an examination, per vagina, and found the os uteri laying on the floor of the vagina, but very slightly open and as hard and unyielding as the mouth of a jug. I could introduce my forefinger, but could not dilate the cervix sufficiently to get two fingers in. Nothing could be felt inside the uterus. There appeared to be only a small amount of a viscid mucus in the cervix. I placed a tampon of sponge, saturated with Ext. Belladonna, in the cervix, but after an hour was unable to dilate the organ in the least. Early on the morning of the 15th I was sent for to attend this cow. On arrival at the ranch the foreman informed me that she had been straining a little at times since my examination on Tuesday. On going to her stall I found she had only a moment before aborted a dead fœtus. On examination the fœtus was found entirely enveloped by the membranes, and on opening these the *liquor amnii* was found to be quite absorbed, and the membranes which were very pale, thin, friable, and quite structureless, fitted very closely to the fœtus. The fœtus, with the exception of absorption of the eyes, was not decomposed at all. It was somewhat mummified, being only slightly moist from its contact with the membranes. From its size it appeared to be about three and a half to four months old. On examining the cow the os uteri was found to be fairly dilated, but still appeared to be quite thick ; the uterus was somewhat contracted. A small quantity of fœtid and disorganized pus and mucus was discharged from the uterus, though but little liquid of any kind, and no blood, had been expelled with the fœtus.

The uterus was at once thoroughly washed out with two or three gallons of a warm solution of Potassii Permanganate. The cow appeared not to suffer the least shock from this abortion, in fact, so far as could be observed, she was in perfect health. Much care is taken on this ranch to keep an accurate record of dates of breeding of cows, and by reference to this record this cow was served by the bull, the last time, on December 15th, 1890, just thirteen months prior to this abortion. There was no possibility of her having been accidentally served during the interim, hence assuming that this fœtus was four months old, she must have been carrying it dead for nine months without any apparent injury to her health. This abortion was, no doubt, caused directly by my irritation of the cervix on the 12th inst., three days prior, but had not this occurred it is quite uncertain how long she might have carried this fœtus. This cow had not, at any time, shown signs of calving, having been dry for some time, though for a couple of weeks, about the latter part of October, 1891, she had a slight discharge of a glairy mucus per vagina.

She is now eleven years old and has always been a regular breeder. It is not known that any accident happened her this time to cause this peculiar trouble. Fleming, in his Veterinary Obstetrics, pages 193–195, records several cases of "fœtal retention," but in most, if not all of these cases, the animals were destroyed or died.

Colorado Springs, Col.

